# A Duplex PCR-Based Assay for Measuring the Amount of Bacterial Contamination in a Nucleic Acid Extract from a Culture of Free-Living Protists

**DOI:** 10.1371/journal.pone.0061732

**Published:** 2013-04-12

**Authors:** Alan O. Marron, Michael Akam, Giselle Walker

**Affiliations:** 1 Department of Zoology, University of Cambridge, Cambridge, United Kingdom; 2 Department of Botany, University of Otago, Dunedin, New Zealand; Indian Institute of Science, India

## Abstract

**Background:**

Cultures of heterotrophic protists often require co-culturing with bacteria to act as a source of nutrition. Such cultures will contain varying levels of intrinsic bacterial contamination that can interfere with molecular research and cause problems with the collection of sufficient material for sequencing. Measuring the levels of bacterial contamination for the purposes of molecular biology research is non-trivial, and can be complicated by the presence of a diverse bacterial flora, or by differences in the relative nucleic acid yield per bacterial or eukaryotic cell.

**Principal Findings:**

Here we describe a duplex PCR-based assay that can be used to measure the levels of contamination from marine bacteria in a culture of loricate choanoflagellates. By comparison to a standard culture of known target sequence content, the assay can be used to quantify the relative proportions of bacterial and choanoflagellate material in DNA or RNA samples extracted from a culture. We apply the assay to compare methods of purifying choanoflagellate cultures prior to DNA extraction, to determine their effectiveness in reducing bacterial contamination. Together with measurements of the total nucleic acid concentration, the assay can then be used as the basis for determining the absolute amounts of choanoflagellate DNA or RNA present in a sample.

**Conclusions:**

The assay protocol we describe here is a simple and relatively inexpensive method of measuring contamination levels in nucleic acid samples. This provides a new way to establish quantification and purification protocols for molecular biology and genomics in novel heterotrophic protist species. Guidelines are provided to develop a similar protocol for use with any protistan culture. This assay method is recommended where qPCR equipment is unavailable, where qPCR is not viable because of the nature of the bacterial contamination or starting material, or where prior sequence information is insufficient to develop qPCR protocols.

## Introduction

A major obstacle to molecular research on free-living, heterotrophic protists is their mode of nutrition. Most such species eat bacteria and therefore require either a source of live bacteria to feed on, or a culture medium containing an axenic substitute. Determining the specific nutritional requirements of heterotrophic protists is a difficult process, meaning that axenic cultures are difficult to obtain for novel experimental species prior to molecular work. Cultures will therefore be required to contain bacteria (*e.g.* [1]), and DNA and RNA extracted from these cultures will almost always contain some bacterial contamination. The amount of contamination varies from species to species and from culture to culture, but it can be up to 99% [2].

A possible solution to this problem is to create a monoxenic culture containing the protistan species plus one unique strain of prey bacteria, and then to separate DNA obtained on the basis of GC content [3]. Developing such methods is a lengthy and difficult process. This may not be feasible when working on a novel species because of constraints on time and funding. Instead ways of purifying cultures to remove as much bacterial contamination as possible have been developed. This includes treatment with antibiotics [4] or separation by density gradient [5]. In order to determine the effectiveness of purification techniques for removing bacterial contamination it is necessary to have a method to quantify the relative proportions of eukaryotic and prokaryotic material after purification.

Testing the eukaryotic purity of cultures has traditionally relied on cell counts by light microscopy to estimate the relative numbers of eukaryotic or bacterial cells before and after various purification methods [4]. This method is often confounded by stratification in the culture flask due to oxygen levels or clumps of bacteria. Alternative methods involving flow cytometry have been developed for larger-scale quantification of the numbers of cell types [6], [7]. These quantification methods assume that relative nucleic acid yields correspond directly to relative cell numbers. This is not always the case, due to differential cell lysis reducing the relative yield of protistan DNA or RNA.

An alternative method to measure bacterial contamination of DNA or RNA samples is to use the polymerase chain reaction (PCR). This may be used as a purely qualitative detection method, where the amplification of products by prokaryote-specific primers indicates the presence of bacterial contamination. Real-time or quantitative PCR (qPCR) can be used to provide a quantitative measurement of bacterial contamination [8], [9]. However qPCR techniques require the use of specifically-designed primers [10], prior knowledge of the molecular biology of the target species (to design appropriate controls [11]) and precise quantification of the amount of material used for nucleic acid extraction [8]. This may not be possible when experimenting with novel heterotrophic protist species, due to factors such as a diverse bacterial flora or starting material variability.

Here we describe a basic PCR-based method for assaying the relative amounts of protistan and bacterial DNA or RNA in an extract from a single-species culture of loricate choanoflagellates. The basis for this method is that the 18S subunit ribosomal RNA gene is only found in eukaryotes, while the 16S subunit ribosomal RNA gene is characteristic for marine prokaryotes (prey bacteria), and as such the prokaryotic signal can be easily distinguished from organelle (mitochondria or chloroplast) signal. By design of appropriate primers and performing a duplex PCR the relative percentage of prokaryotic contamination in eukaryotic material can be quantified. Analysis of the duplex-PCR amplification products can provide relative quantification of bacterial contamination even without complete knowledge of bacterial diversity or starting material content. This assay can then be applied to various culture purification methods to test their effectiveness.

## Results

**Figure 1:**
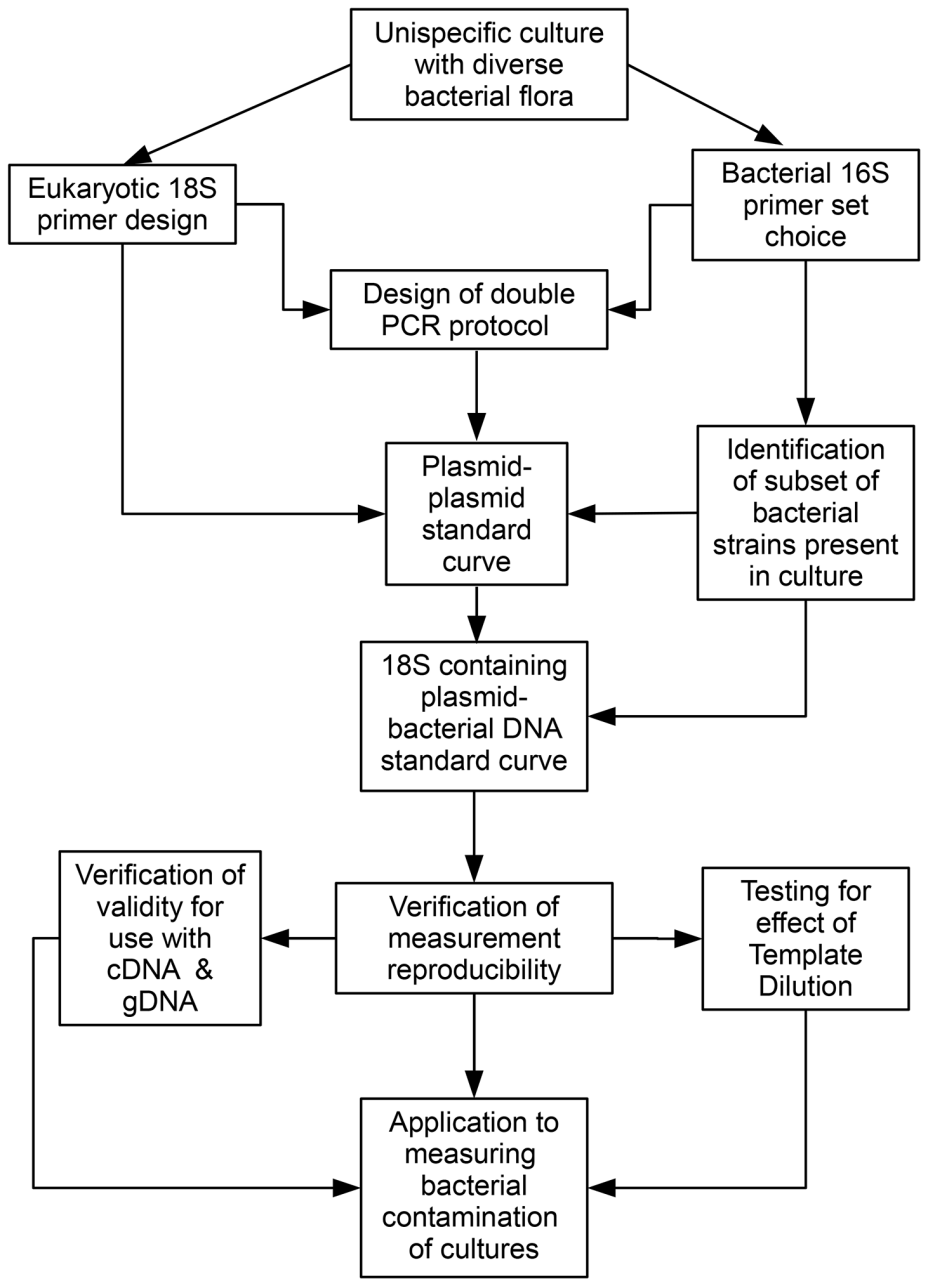
Outline procedure for development of the duplex PCR-based assay. These steps were used to measure the levels of bacterial contamination in nucleic acid extracts from cultures of the loricate choanoflagellates *Stephanoeca diplocostata* and *Diaphanoeca grandis*.

Figure 1 shows the overall procedure used to develop and verify our duplex PCR assay. The principle of the assay is that, at saturation, the relative amount of each of the two amplified products generated in the duplex PCR will depend on the relative concentrations of the two rRNA target sequences in the template, and this provides a measure of the relative amounts of eukaryotic and prokaryotic material in the original nucleic acid sample.

### Standard Curve

In order to establish the validity of the principle behind this assay, it was necessary to perform trials on templates of known concentration, and from these results construct a standard curve. A standard curve then allows calculation of how the relative brightness of the 18S and 16S bands relates to the concentration of the target sequences in the PCR template.

**Figure 2:**
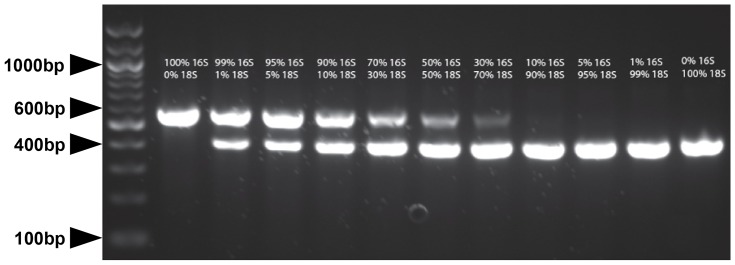
Assay Template Composition versus Relative Band Brightnesses for the Linearized Plasmid Mixture Series. The agarose gel shows the amplification of PCR products from a linearized plasmid-only assay series. The percentages given correspond to the percentage of the 2 µl DNA template volume that was comprised of 4 ng/µl *Marinomonas* sp. 16S or *D. grandis* 18S target sequence-containing linearized plasmid. The relative intensity of the relevant band (550 bp for 16S, 400bp for 18S) is observed to vary according to the percentage of target sequence in the template DNA. It should be noted that the percentages quoted concern weight per volume, however the differences in product sizes (400 bp vs. 550 bp) mean that the molar ratios (i.e. number of DNA molecules) are 1.375∶1 18S∶16S by concentration. The 100 bp ladder (Invitrogen) is shown in the first lane with the 1000 bp, 600 bp, 400 bp and 100 bp markers noted.

Initially this was done by mixing known quantities of two separate linearized plasmids containing either the relevant 16S or 18S sequences, and using these as artificial templates for the duplex PCR assay. The results from this plasmid-only assay series (see figure 2) confirm that the relative brightnesses of the 16S and 18S bands are related to the composition of the template. The greater the amount of respective target sequence the brighter the relevant band is, when expressed as a percentage of the total band brightnesses.

A series of simulated culture templates were constructed using known amounts of *Marinomonas* sp. gDNA as the sole source of 16S target sequence and linearized plasmid as the sole source of choanoflagellate 18S target sequence. The aim of this assay series was that the non-target background sequence (i.e the majority of the bacterial gDNA) would better mimic a real-life DNA template. This would therefore produce a more biologically representative set of assay results for constructing a standard curve.

**Figure 3:**
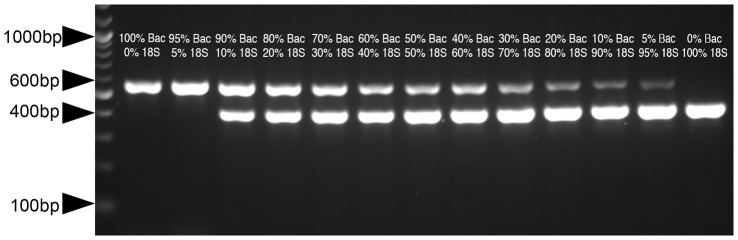
Assay Template Composition versus Relative Band Brightnesses for a Simulated Culture Series. The simulated culture assay series demonstrates that the relationship between assay template composition and relative band brightness still holds even in the presence of background non-target DNA. The agarose gel shows the amplification of PCR products from a simulated culture assay series. The percentages given correspond to the percentage of the 2 µl template DNA that was bacterial *Marinomonas* sp. gDNA or *D. grandis* 18S target sequencing-containing linearized plasmid. Both the bacterial gDNA and linearized plasmid were diluted such that the estimated concentration of 16S or 18S target sequence was equal at 0.1 ng/µl. The 100 bp ladder (Invitrogen) is shown in the first lane with the 1000 bp, 600 bp, 400 bp and 100 bp markers noted.

**Figure 4:**
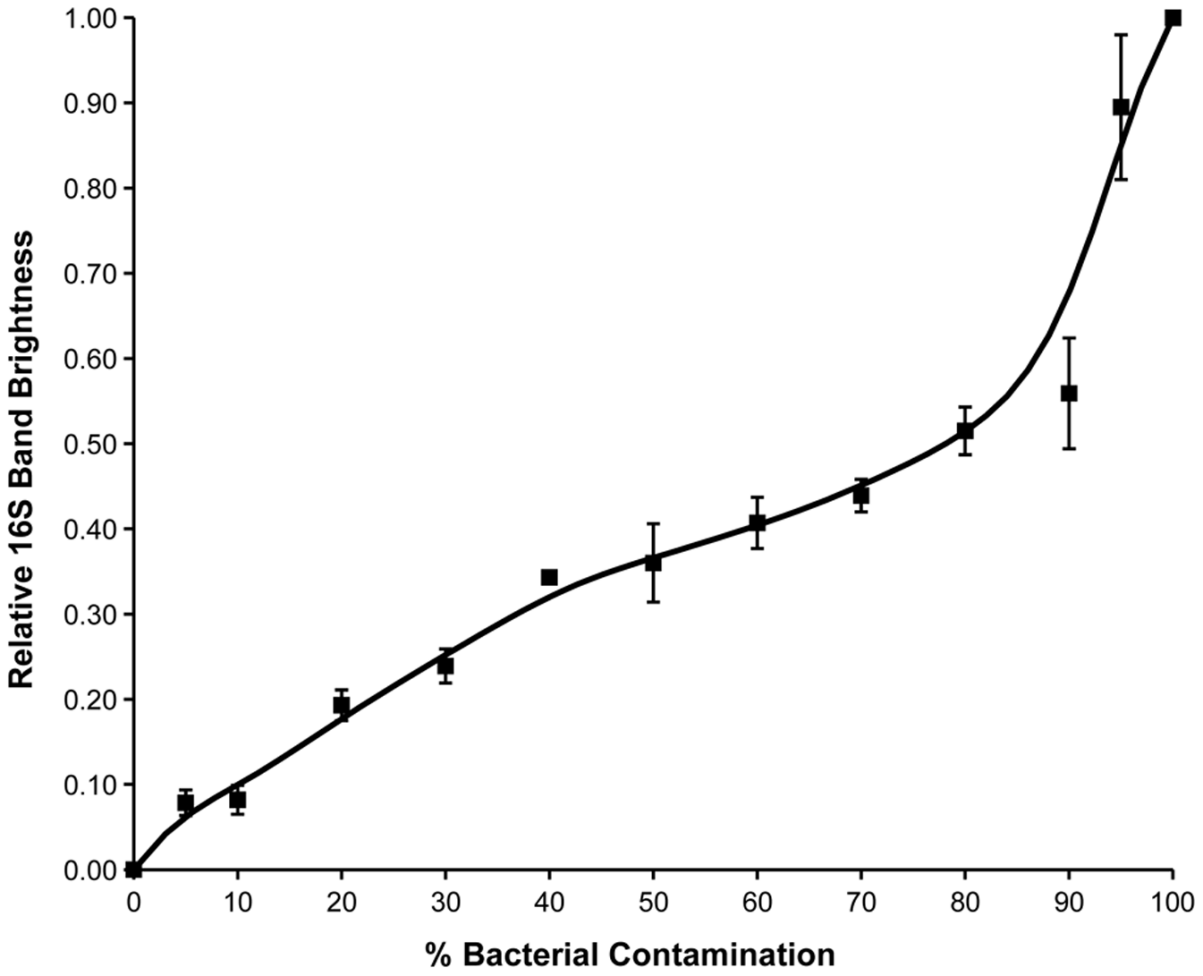
Assay Standard Curve for use in Quantification of Bacterial Contamination of Loricate Choanoflagellate Cultures. The relative 16S band brightness is here plotted against percentage of bacterial 16S target sequence in the template DNA. The error bars at each point show ± standard error. This graph can be used as a standard curve to determine the relative levels of bacterial contamination in DNA samples. It may also be used in conjunction with absolute readings of DNA concentration to provide the basis for absolute quantification of the bacterial and choanoflagellate content in the original nucleic acid sample.

Figure 3 shows the gel displaying the amplification products of a replicate of this duplex-PCR assay. The change in signal intensity for the bands varies in a similar fashion to the plasmid-only assay across the template composition series, indicating that background DNA has little effect on the dynamics of the assay reactions. Figure 4 is a standard curve of mean relative 16S band brightness versus percentage bacterial gDNA present in the template. As this standard curve was calibrated to estimated target sequence amounts in 100 ng of template, applying it for comparison of real-life culture assay results requires a template containing *ca.*100 ng of gDNA or cDNA per assay to minimize dilution effects (see below).

### Statistical Verification of the Assay

**Figure 5:**
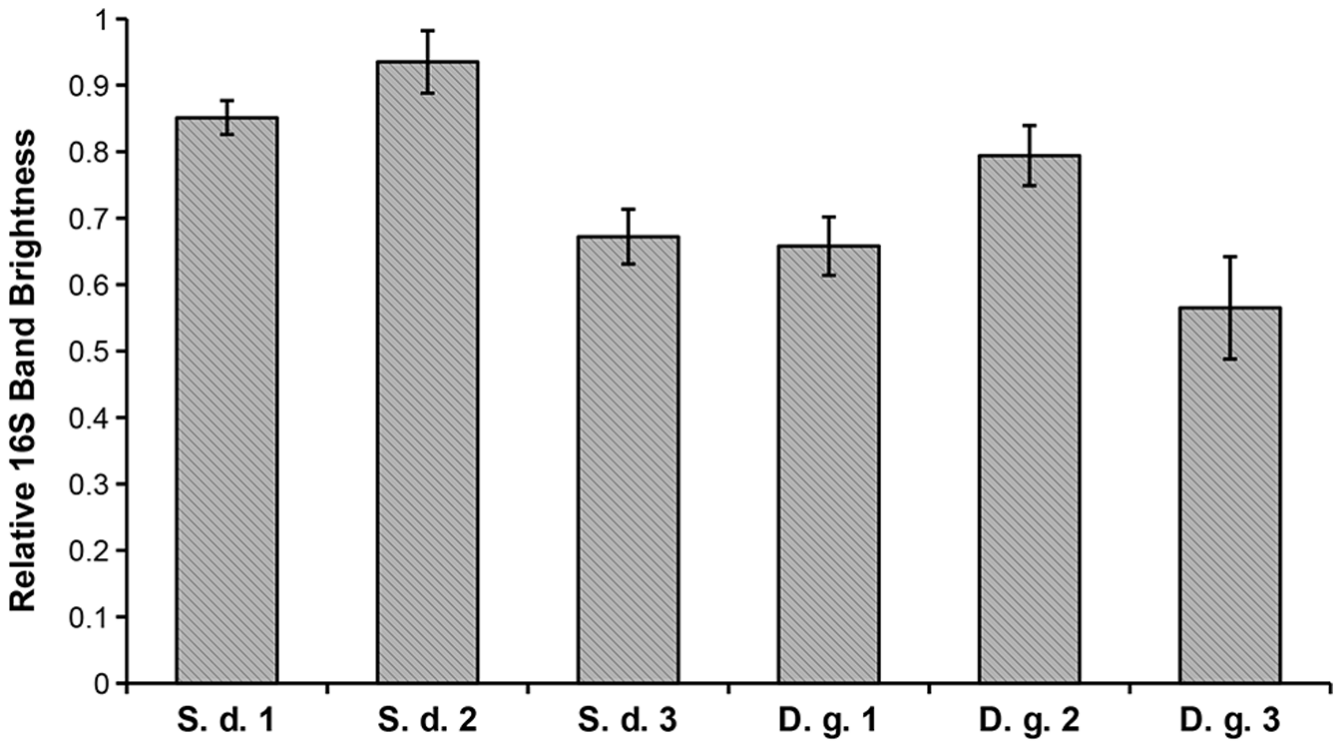
Statistical Verification of Repeatability of Assay Measurements. Shown are mean relative brightness measurements of the 16S band (bars showing ± standard error) from triplicate assays performed on six monospecific choanoflagellate cultures; three *S. diplocostata* (S.d. 1–3) and three *D. grandis* (D.g.1–3). The values are significantly different (F_5,57_ = 10.48, P<0.001) between the cultures such that measurements are repeatable and representative of the culture they are taken from.

To verify the suitability of this assay for real-life cultures, assays were performed on six different choanoflagellate cultures grown under similar conditions. Preliminary observations by cell counting show that such cultures typically vary in the yield of choanoflagellates. Triplicate assays were performed on each culture to test for repeatability and specificity of the assay measurements for an individual culture. The mean values with standard error bars of the relative brightness of the 16S band for each of six cultures are given in figure 5. The REML (residual maximum likelihood) analysis found that there was a significant difference between cultures (F_5,57_ = 10.48, P<0.001), indicating that the cultures are not all drawn from a single, randomly distributed population. This means that the variability of measurements from a single culture is small enough such that measurements from each culture are characteristic for that culture and represent actual biological variation. Therefore the use of a duplex PCR with these 18S and 16S primer pairs can be used to determine differences in the proportion of eukaryotic gDNA extracted from a specific choanoflagellate culture.

### Use for cDNA

**Figure 6:**
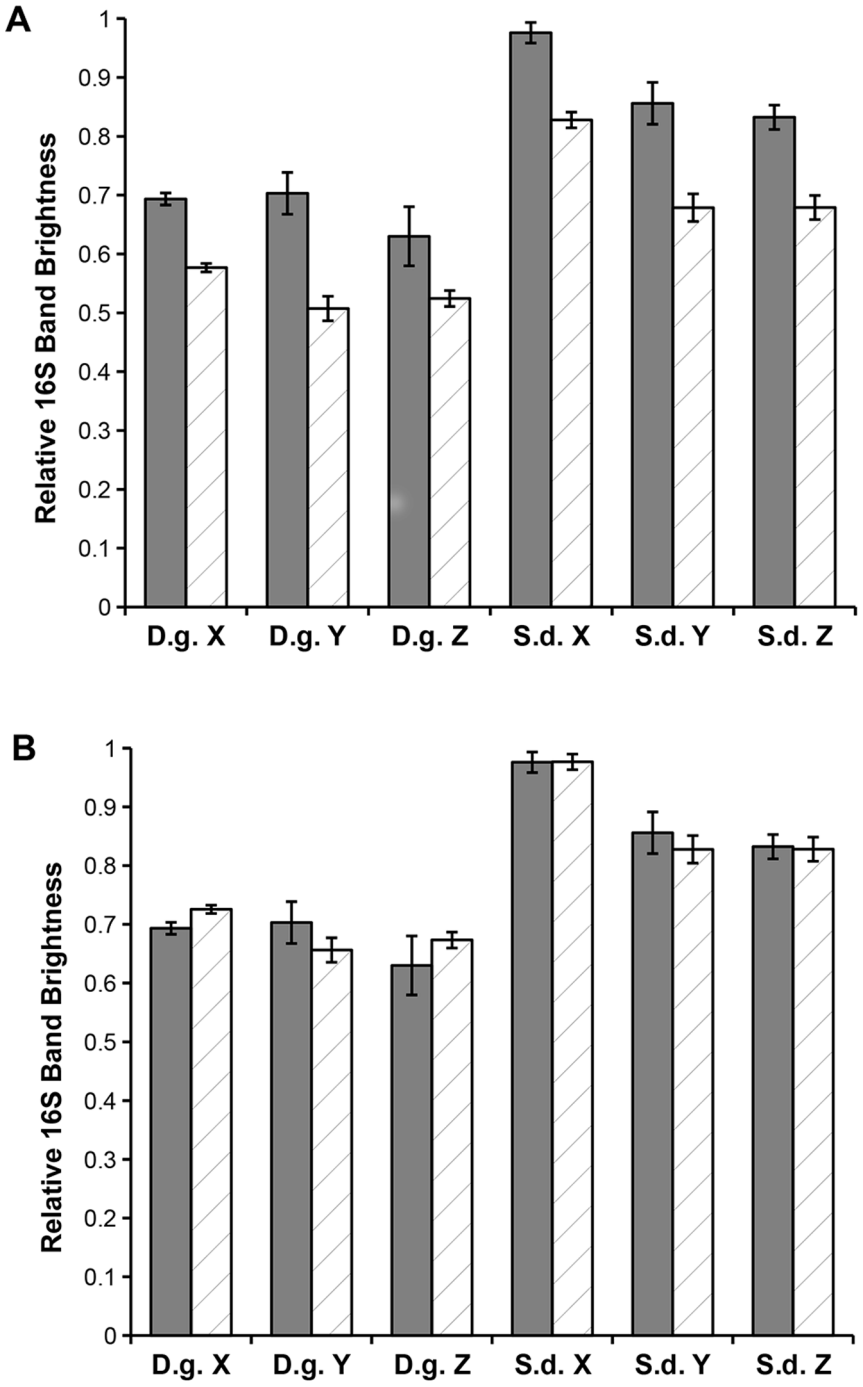
Assay Measurements from cDNA Template reflect Original Culture Composition. A The unadjusted cDNA results show a significant difference between gDNA and cDNA results (F_5,57_ = 13.83, P<0.001). B Applying a 0.149 adjustment to the cDNA results show no significant difference (F_5,57_ = 1.06, P = 0.403) between gDNA and cDNA results. The bars show mean relative 16S band brightness (±standard error) for either gDNA (grey bars) or cDNA (hatched bars) template from three *D. grandis* cultures (D.g. X, Y, Z) and three *S. diplocostata* cultures (S.d. X, Y, Z).

To examine whether the assay was suitable for measuring bacterial contamination of RNA samples, assay measurements of gDNA and cDNA produced from the same source culture were compared. Figure 6A shows the mean assay measurements for the relative brightness of the 16S band from both cDNA and gDNA from each culture. The REML analysis found a significant but consistent difference between cDNA and gDNA relative band brightness (F_5,57_ = 13.83, P<0.001). Therefore measurements taken using extracted gDNA in the assay are not directly applicable to cDNA. The cDNA assay results have greater 18S signal and less 16S signal compared to the gDNA with a mean difference of 0.149 relative brightness. Applying this 0.149 conversion factor to the measurements (see figure 6B) and statistical reanalysis shows no significant difference between cDNA and gDNA (F_5,57_ = 1.06, P = 0.403). This correction factor is applicable to both *D. grandis* and *S. diplocostata* samples. If it is assumed that random hexamer priming in the reverse transcription reaction has no bias to prokaryotic or eukaryotic material, then this reflects the composition of the RNA extract from which the cDNA was synthesised.

### Dilution Effect

**Figure 7:**
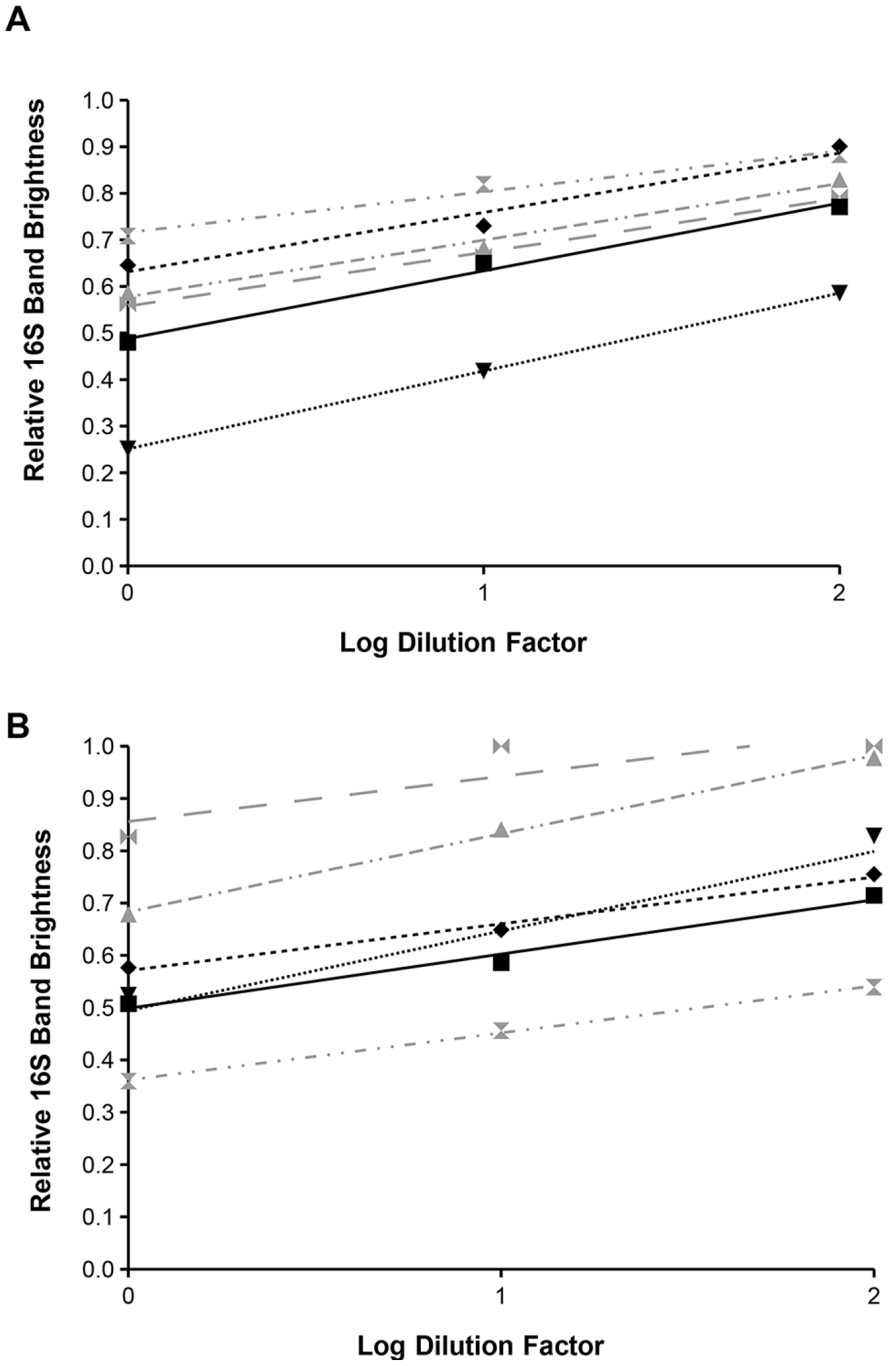
Assay Dilution Series. The dilution series shows a linear relationship between template concentration and relative 16S band brightness for gDNA (A) and cDNA (B). Relative 16S band brightness is plotted against log_10_ dilution factor. Symbol shape and line style correspond to nucleic acid samples from individual cultures. Grey symbols and lines indicate *S. diplocostata*, black symbols and lines indicate *D. grandis*. Trend lines fitted by regression analysis found an average slope of 0.12±0.01 across all samples. Trend lines in all cases had r^2^ >0.9, with the exception of one dilution set which showed saturation (relative 16S band brightness = 1) after 10-fold dilution (in this case r^2^ = 0.75).

The effect of DNA template concentration on relative band brightness was examined by serial dilution of templates (see figure 7). Both *D. grandis* and *S. diplocostata* assays show a similar pattern whereby the relative brightness of the 16S band increases as the DNA template becomes more dilute. This dilution effect is observed for both gDNA (figure 7A) and cDNA (figure 7B) and is consistent irrespective of the concentration or relative band brightness of the undiluted template. Regression analysis calculated well-fitting (average r^2^ = 0.94) linear relationships between relative band brightness and dilution factor, such that the relative 16S band brightness increases by an average 0.12 (±0.01 standard error) per 10-fold dilution of template. This relationship is consistent for both species (*S. diplocostata* = 0.11±0.01, *D. grandis* = 0.13±0.01), and for cDNA (0.11±0.01) and gDNA (0.13±0.01). The consistent linear relationship between relative band brightness and template concentration means that, at a given template amount, independent assay results are comparable.

### Application of assay to Culture Purification

**Figure 8:**
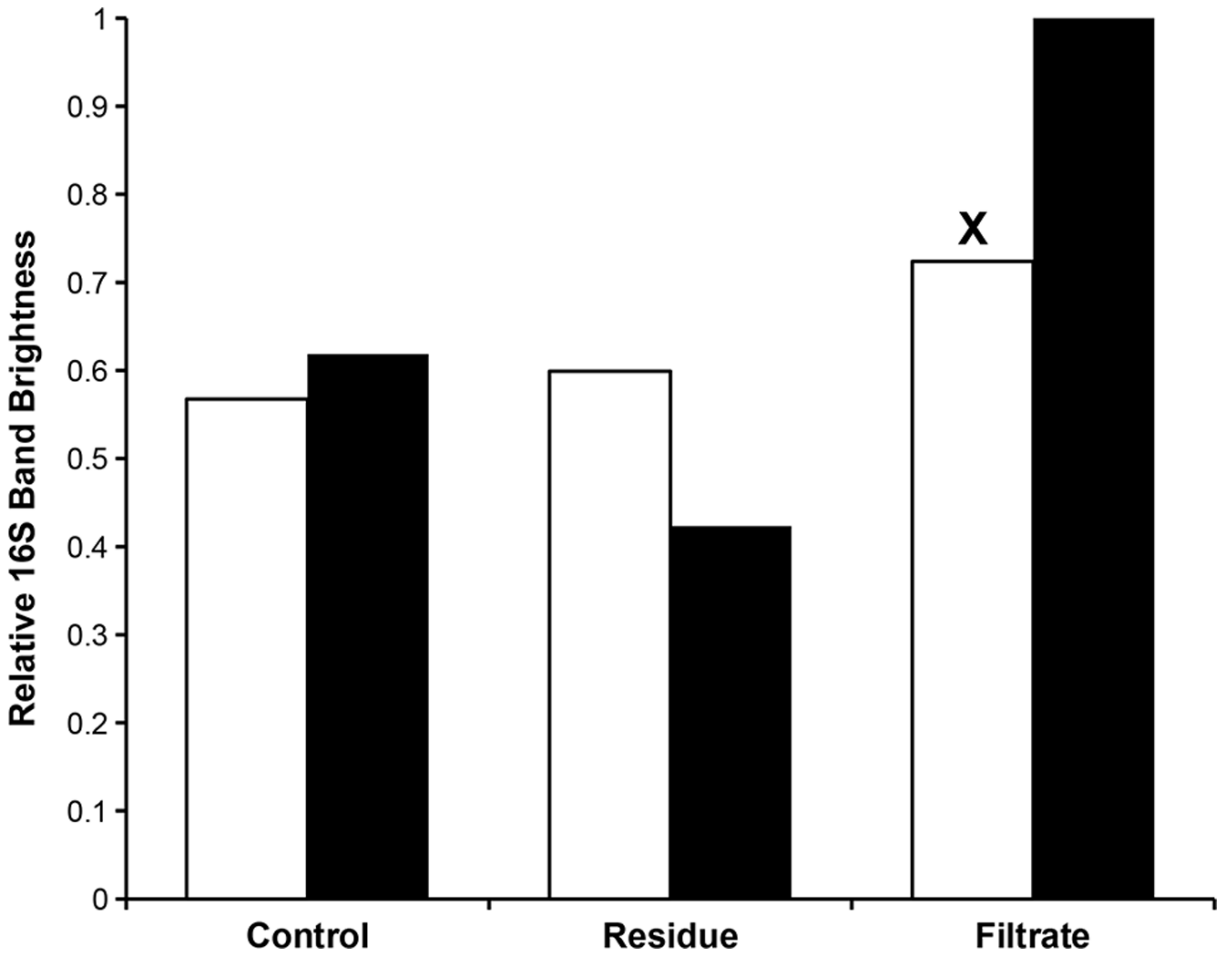
The Effect of Antibiotic and Filtration Treatment on the Relative Bacterial Contents of Choanoflagellate Cultures. White bars are assay results from *S. diplocostata*, black bars are assay results from *D. grandis.* For *D. grandis* the treated residues show a lower bacterial content than the unfiltered and untreated control. For *S. diplocostata* the treated residue has a slightly higher bacterial content than the control samples. The *D. grandis* filtrate contains only bacterial signal, whilst for *S. diplocostata* a relative 18S band brightness of 0.28 is present (X), indicating that choanoflagellates are present in the filtrate.

One potential application of the assay is to measure and compare the effectiveness of different purification methods for reducing prokaryotic contamination in cultures. The assay results (figure 8) found that, for *D. grandis* cultures, a 36 hour antibiotic treatment followed by filtration purification produced a reduction in relative 16S band brightness of 0.2, from 0.62 to 0.42. Application of the standard curve finds that this corresponds to a threefold enrichment of choanoflagellate versus bacterial DNA, from 13% to 39%. For *S. diplocostata*, the combined antibiotic and filtration treatment was found to have no impact on the amount of bacterial contamination, with the relative 16S band brightness of the residue being slightly higher than that of the original sample. Assaying of the filtrates found that while the *D. grandis* filtrate is composed solely of bacteria (relative 16S band brightness = 1), a notable 18S signal is present in the *S. diplocostata* filtrate (X, figure 8), explaining why the purification method was ineffective for this species.

## Discussion

Figure 1 shows the procedures used to develop this duplex PCR-based assay for quantification of bacterial contamination in loricate choanoflagellate cultures. Once established, this assay provides a simple, fast and cheap assay needing no specialized equipment beyond that required for carrying out standard polymerase chain reactions. The assay allows multiple samples from many different cultures to be assayed in parallel, and provides a means for relative quantification (for example to compare culture purification methods). It can also be used as the basis for absolute quantification of eukaryotic material in a sample, by applying total sample concentration measurements to a calibrated standard curve constructed using certain biological assumptions. The assay method described here provides a template for application to other heterotrophic protist cultures, opening up avenues for molecular biology and genomics in novel protist species, as well as refinement of quantification and purification procedures in currently- researched non-model species.

### Considerations for Assay Design

The methods outlined for this assay may also be redesigned for use with other protistan cultures to measure bacterial contamination. It is advisable to design primers to the 18S SSU rRNA gene for several reasons. The 18S gene is only found in eukaryotes and therefore all amplified product signal can be attributed to a protistan nuclear source, provided the primers have been tested for non-specific priming. SSU rRNA genes usually have multiple copy numbers and are highly expressed allowing for easy amplification from either gDNA or RNA. The 18S gene is widely used in phylogenies, meaning that sequences are available for the design of suitable primers for a wide range of species [12]. Primer sets are available that will specifically amplify 18S sequences from certain groups (*e.g.* fungi, [13]), as well as universal eukaryotic 18S primer sets [14].

The 16S gene has similarly been used for prokaryotic identification and phylogeny. Universal 16S primers developed for environmental sampling allow amplification from a range of bacteria, overcoming difficulties related to bacterial diversity in cultures. In this case the universal primers were originally used in the study of temperate marine environments [15] similar to the one from which the choanoflagellates in this study were first isolated. The stable culturing and clonal axenic methods used to produce new loricate choanoflagellate cultures were predicted to retain the original marine bacterial flora of the cultures.

It should be noted that the application of this assay to general cultures is dependent on the 16S universal primer set used amplifying the target sequence from all bacteria present equally as well as the test species (in this case *Marinomonas* sp.). For this reason it is important that the primer set chosen is appropriate to the bacterial community and sensitive enough to amplify the majority of bacterial species in the culture. If a large proportion of the bacteria have rRNA sequences poorly amplified by the primers (or not amplified at all) then the assay will underestimate the prokaryotic percentage and overestimate the eukaryotic content. If the cultures are predicted to contain significant amounts of archaea, then archaeal 16S-specific primers [16] will be required; either for use in a triplex PCR to measure total prokaryotic contamination, or in the design of a secondary PCR assay to measure archaeal contamination only.

Matched reaction dynamics of the primer sets ensure that the duplex PCR is repeatable and only dependent on the template composition. As product size has a large influence on reaction dynamics [17], primers should be designed to amplify sequences of similar length, with the limitation that they must still be distinguishable visually on an agarose gel. A size difference of approximately 200bp is considered suitable from the findings here. Due to highly degenerate primer pairs having reduced priming efficiency compared to non-degenerate primers, the primer sets should be of as low degeneracy as possible. In addition, the PCR protocol should be designed for use with the more degenerate of the primer sets. Low degeneracy primers are preferable because low temperature protocol suitable for highly degenerate primers may lead to random non-specific priming by the eukaryotic primer set.

The composition of the reaction mix may have to be adjusted to compensate for primer efficiency. In an idealized duplex PCR, neither target sequence is preferentially amplified with respect to the other, and the relative initial amounts of the products formed are a direct function of the percentage of the template DNA. Following this, amplification (and therefore signal strength) is rate-limited by competition between the products for Taq polymerase and dNTPs. It may be necessary to adjust the ratios of the primers if preferential amplification of one product over the other does occur. Primer ratios may also be adjusted to improve sensitivity for template composition. For example if high levels of prokaryotic contamination were present then the relative amount of 18S primers used for the assay should be increased. This would improve assay sensitivity by causing small absolute changes in eukaryotic template to appear as a large changes in assay signal (as was the case for the loricate choanoflagellate cultures, see figure 3).

Assay results are characteristic for each culture, however a linear relationship is observed between relative band brightnesses and template concentration. For both cultures tested here, lower amounts of template caused an apparent increase in the amount of prokaryotic signal while lowering the relative amount of choanoflagellate signal. The effect of this is such that a 10-fold dilution would lead to a 12% overestimation of the amount of bacterial signal (see figure 7). The reasons for this dilution effect are unclear. It would be expected that a reduction in template amount would promote amplification from the lower degeneracy primers (18S) and of the shorter amplicon (18S); however the reverse was found in this case (i.e. greater amplification of the 16S amplicon). It is therefore crucial that semi-quantitative comparisons of bacterial contamination between cultures are carried out using equal amounts of gDNA or cDNA template. Furthermore, it is important that the template concentrations used to construct the standard curve are biologically relevant to the target sequence concentrations expected of real nucleic acid samples extracted from the cultures. In this case we used target sequence amounts corresponding to ∼100 ng of normal template. It should be noted that this assay is robust to minor dilution effects, as small differences in concentration may be masked by the minor natural variability observed in assay results (figure 5) and standard curve measurements (figure 4).

### Applications for Purification Methods

Purification methods such as antibiotic treatment and filtration are often applied to protistan cultures to reduce bacterial contamination [3], [4]. The assay described here can be used to test the effectiveness of purification methods by comparing the percentage of eukaryotic material recovered before and after purification of a culture. While the total amount of nucleic acid recovered may be lower following purification, the overall eukaryotic proportion may be higher, a conclusion that could not be determined solely by measuring nucleic acid concentration.

In the example presented here (see figure 8), a 36-hour antibiotic treatment followed by filtration through a 10 µm mesh was effective in enriching the relative choanoflagellate content of *D. grandis* cultures approximately threefold. In contrast, prokaryotic contamination levels were not reduced in *S. diplocostata* cultures. The assay here provides further analysis of the reasons for this, as the assayed filtrate sample contains choanoflagellate signal (X, figure 8). This suggests that *S. diplocostata* cells were passing through the filter mesh pores, while the larger *D. grandis* cells [18] remained in the residue. Detailed light microscope investigations found that antibiotic treatment produces aloricate *S. diplocostata* cells that could then pass through the pores in the filter mesh (A. Marron, pers. observ.). In this case the PCR-based assay provided a faster and more comprehensive analysis of the effectiveness of this purification technique than light microscope-based observations alone.

The standard curve can also be used for absolute quantification of DNA or RNA extracted from a culture. The estimate of the percentage of eukaryotic material from the assay result can be used in conjunction with total concentration measurements (from instruments such as a Nanodrop Spectrophotometer or Agilent Bioanalyser) to determine the amount of choanoflagellate material present in a nucleic acid sample. It is important to note that using the standard curve for absolute quantification is based on two assumptions.

Firstly, the copy number of the 16S rRNA gene in *Marinomonas* sp. is assumed to be representative of the whole bacterial flora. Although rRNA gene copy number is highly variable across prokaryotes, eight 16S genes per genome is intermediate between the maximum (15) and minimum (1) values reported in the literature [19]. For the application of the assay procedure to other protistan cultures, the copy number should be adjusted to reflect the bacterial species from which the standard curve DNA was isolated (see Methods).

The second assumption is that the target sequence comprises the same percentage of the eukaryotic and bacterial genome (or transcriptome). In the case of the standard curve constructed here, this is approximated to 0.001%; however an accurate figure is difficult to calculate without having a fully sequenced genome of the loricate choanoflagellate species being investigated. It is more likely that the 18S rRNA sequence would make up a smaller percentage of the total eukaryotic genome than the 16S rRNA target sequence would of the bacterial genomes. In this case the standard curve would underestimate the eukaryotic proportion of the DNA or RNA sample, providing a lower bound for the quantity of eukaryotic nucleic acid collected.

Absolute quantification of eukaryotic content is necessary for sequencing purposes, where there are minimum quantities of starting material required for sequencing technologies. Bacterial contamination is a major obstacle in genome sequence data analysis and gene model construction [3]. A possible solution is transcriptome sequencing [20], where RNA can be enriched for polyadenylated (*i.e.* eukaryotic) material. However it is crucial to have sufficient eukaryotic RNA in the samples for cDNA library construction. For both situations the duplex PCR assay outlined here would provide a suitable starting point to determine the total amount of DNA or RNA extracted from the protistan species of interest.

### Advantages of Assay over other Quantification Methods

While direct observations of protist cultures can be used to estimate the relative numbers of eukaryotic and prokaryotic cells, the accuracy of these counts is restricted by the sampling error and variations in bacterial morphology and behaviour (*e.g.* clumping, swimming). Larger-scale observations, such as flow cytometry, are still limited by the assumption that nucleic acid recovery is directly related to cell numbers, which may not be valid in cases where protistan cells are resistant to lysis, or where protist RNA or DNA is prone to degradation. Quantitative PCR (qPCR) can be used to investigate prokaryotic contamination of nucleic acid samples extracted from protist cultures, and can provide both relative and absolute quantification data [21], [22]. This method has been applied successfully to samples with intrinsic prokaryotic contamination such as plant and animal tissue containing intracellular mycoplasmas [23] and duodenal flow samples containing both protozoa and bacteria [24].

However, qPCR has several drawbacks that make it unsuitable for use with novel protistan cultures such as loricate choanoflagellates. Optimum qPCR conditions require the primer sets to be used to be non-degenerate and to amplify products 200–400 bp long [21]. For a culture containing a diverse and incompletely characterised bacterial community, design of suitable primer pairs is non-trivial and in some cases may be impossible. For RT-qPCR normalization is usually required, which is done by measuring the amplification of a third PCR product [11], [22]. Novel protist species may not have the necessary data to design a suitable normalization reaction. Non-normalized qPCR-based assays are based on using known weights of starting material (*e.g.* leaf tissue, [8] for nucleic acid extraction, so that prokaryotic contamination can be quantified relative to a fixed amount of eukaryotic material. This is not always possible for protist cultures, especially those where cell numbers may be difficult to count accurately or where bacterial cells will comprise a significant portion of the starting material weight. qPCR requires specialist equipment, training and analysis procedures, which may not be available for use in all laboratories, and their installation and development is an expensive procedure.

In contrast, the assay described in this paper can be carried out using traditional PCR apparatus and only requires training in standard molecular biological techniques, such as plasmid-based cloning, restriction digests and DNA quantification. Using the procedures outlined in this paper, the duplex assay method can be adapted to any protistan culture for which 18S sequence data and suitable universal bacterial primers are available. It provides a simple, flexible and relatively inexpensive means of determining bacterial contamination for use with non-model protistan species, where difficulties such as a diverse bacterial flora, varying nucleic acid yields and insufficient prior sequence data prevents the use of traditional methods for measuring the levels of bacterial contamination. The novel use of this type of duplex PCR-based assay with heterotrophic protist cultures can then be applied to testing purification methods, establishing mono- or axenic cultures and to quantification and verification of nucleic acid samples intended for genomic sequencing.

## Materials and Methods

### Cultures

Mono-eukaryotic cultures of *Diaphanoeca grandis* Ellis 1930 and *Stephanoeca diplocostata* Ellis 1929 were obtained from Barry Leadbeater, (University of Birmingham, UK) who isolated these cultures. The culture media for both species consisted of artificial seawater made using Tropic Marin salts (Dr. Biener Aquarientechnik, Wartenberg Germany), which was vacuum-filtered through a 0.22 µm Steriop GP Express Plus filter (Millipore, Massachusetts U.S.A.) and autoclaved. An autoclaved sterile rice grain was added to the culture medium in order to provide food for the bacteria present in the culture. Cultures were grown in sterile screw-top bottles (Schott Duran) at 13.5°C. Every 3–4 weeks cultures were split to form new cultures. The cultures were monitored using a light microscope to ensure that the choanoflagellate populations were healthy and that there was no contamination. Fungal contamination was tested by using PCR with universal fungal primers [13] on a template of total culture gDNA extracted using CTAB buffer [25].

### Eukaryotic Primer Design and Verification

18S subunit ribosomal RNA gene sequences from *Diaphanoeca grandis* and *Stephanoeca diplocostata* were obtained from the EMBL/Genbank database. The sequences gi33337666 (C.L. Adams, unpub.) and gi69048564 [26] were used for *D. grandis* while for *S. diplocostata* the sequences gi157780191 [27], gi157780190 [27], gi33337667 (C.L. Adams, unpub.) and gi37359232 [28]. Sequences were aligned using ClustalX 2.0.9 (www.clustal.org) with the aim of identifying conserved regions, to which to design non-degenerate primers (see table 1).

**Table 1:** 18S and 16S primer sequences used in the assaying of loricate choanoflagellate cultures.

18S Forward Primer	TCCAAGGAAGGCAGCAGG
18S Reverse Primer	AGTCCTATTCCATTATTCCATG
16S Forward Primer (357fGC)	CCTACGGGAGGCAGCAG
16S Reverse Primer (907rM)	CCGTCAATTCMTTTGAGTTT

The 16S primers 357fGC and 907rM are taken from [15]. The 18S primers were designed using the sequences listed in the methods section. The primers were designed to two regions of the alignment; 426 bp–442 bp and 832 bp–853 bp. All primer sequences are given as 5′–3′.

The sequences between these two primer regions are disparate enough to differentiate between *D. grandis* and *S. diplocostata*. Biomath Calculator (www.promega.com/biomath) was used to determine the primer melting temperature (57°C for forward primer, 50°C for reverse primer) and the appropriate annealing temperatures for use in the PCR protocol.

These primers were then tested using BLAST [29] to test for their specificity to loricate choanoflagellates only, and not amplify any bacterial sequences, which would lead to an over-estimation of the amount of choanoflagellate material in any template used. When tested against the eubacterial sequence database no sequences were found matching to the combined 18S primer set.

The primers were successfully used on gDNA extracted from single strain cultures of both species to give PCR products of the appropriate length (412bp for *D. grandis*, 420bp for *S. diplocostata*). The amplified sequences were cloned into a plasmid vector using the PGEM-T Easy Vector System (Promega) and Subcloning Efficiency DH5α Competent Cells (Invitrogen). The plasmids were extracted using a Qiaprep Spin Miniprep Kit (Qiagen). Sequencing was carried out by Geneservice (Cambridge, UK). For each species the correct 18S sequences were returned.

### Prokaryotic Primer Design and Verification

Preliminary identification of the bacterial diversity present in the cultures was carried out using the 16S ID method described in [15] that developed primers for investigating marine bacterial communities. Total culture gDNA extracted using CTAB buffer [25] was used as a template, with the same cloning and sequencing protocol as used for the 18S PCR products. In addition serial streaking on LB agar plates grown at 18°C was used to isolate bacterial strains. When uniform colonies were obtained, samples of the bacteria were grown up in LB broth at 18°C and the gDNA extracted using a Genomic DNA buffer set and Genomic Tips 20/G (Qiagen). The sequences amplified by the 16S primers were then used in a BLAST search in order to identify bacterial species. The strains isolated by streaking on LB agar were found to be *Marinomonas* sp. (gi160964597, gi129561851), and *Thalassospira* sp. (gi65941366). The cultures also contained a representative of the Flexibacteraceae (gi187319458), *Colwellia* sp. (gi125719328) and *Antarctobacter* sp. (gi215414345). The diverse flora found by this preliminary investigation meant that design of appropriate non-degenerate primers for qPCR was not possible. The primers given in [15] were used for all further investigations of the bacterial community present in the cultures (see Table 1).

### Assay PCR Conditions

A protocol for a duplex PCR using both 18S and 16S primers on a template of culture gDNA was developed (see Tables 2, 3). In preliminary experiments it was established that equimolar concentrations of all four primers gave an appropriate balance of prokaryotic and eukaryotic amplification over the biologically relevant range of contaminant ratios, as well as providing maximum sensitivity for measuring small changes in choanoflagellate content (data not shown). The duplex PCR samples produced were run on a 1.5% ethidium bromide-agarose gel. The gels were digitally photographed using an AlphaImager gel reader and AlphaEase FC 6.0.0 software (AlphaInnotech). All photographs were taken at 750 ms, 375 ms, 188 ms and 94 ms exposure time at 2.00 aperture in order to adjust for signal saturation. This range of exposure conditions gave clear imaging of both bands and allowed checking for saturation at higher exposures.

**Table 2:** Recipe for the assay PCR mixture.

Component	Volume
10X Reddymix Buffer	2.5 µl
2 mM dNTPs	2.5 µl
Thermoprime Plus	0.2 µl
18S 10 mM Forward Primer	1.25 µl
18S 10 mMReverse Primer	1.25 µl
16S 10 mM Forward Primer	1.25 µl
16S 10 mM Reverse Primer	1.25 µl
Nucleic Acid Template	X µl
ddH_2_O	to 25 µl

Template volumes were 2 µl for the experiments given in figures 2–7. Following establishment of the standard curve all templates comprised 100 ng of gDNA or cDNA. Both the 10X Reddymix Buffer and the Thermoprime Plus taq polymerase were sourced from Thermo Scientific (Abgene UK, Surrey, UK). All primers were synthesised by Sigma-Aldrich Ltd. (Dorset, UK).

**Table 3:** Assay PCR protocol.

**1 cycle:**	
94°C	5 min
**10 cycles:**	
94°C	1 min
65°C (−1°C touchdown per cycle)	1 min
72°C	3 min
**20 cycles:**	
94°C	1 min
55°C	1 min
72°C	1 min
**1 cycle:**	
72°C	10 min

The initial 94°C cycle requires a hot start at 94°C. The protocol is based on that given in [15].

### Digital Analysis

Digital images of gels were analysed using ImageJ v.1.41 (http://rsb.nih.gov/ij). A rectangular selection was taken around the 16S band and 18S band: mean brightness and band area were measured. The mean brightness of the area between the bands was also measured, and this was subtracted from the band brightness measurements to calculate the adjusted band brightness. The intensity for each band was calculated by multiplying the background adjusted brightness by the area of the band. The relative brightness of each band was expressed as a proportion of the combined band brightness.

### Standard Curve Construction

The assay was done using measured amounts of target 18S and 16S sequence with the aim of constructing a standard curve. A preliminary assay was carried out to determine if the relative band brightness varied predictably according to the amount of target sequence within the reaction. This was done using purified PGEM-T plasmid (Promega) containing the target sequence from either *Marinomonas* sp. or *D. grandis*. The plasmid was linearized using Not1 restriction enzyme, which has a single restriction site outside of the target sequence. The linearization mix contained 10U Not1 enzyme (Roche), 1 µl SuRE/cut Buffer H for Restriction Enzymes (Roche) and 960 ng of plasmid DNA. This mixture was incubated at 37°C for 90 minutes. The linearized plasmid was purified using a Qiaquick PCR Purification Kit (Qiagen). The final concentration of linearized plasmid was measured using a ND-1000 Spectrophotometer and ND-1000 v3.3.0 Software (NanoDrop) to determine the purified plasmid concentrations. The samples were then diluted to give equal final concentrations of 18S and 16S target sequence of 4 ng/μl, providing almost equal molar ratios. The linearized plasmid-based assay test was conducted by performing the assay on mixtures containing 8 ng of template, which in itself contained various percentages between 0% and 100% of 16S and 18S sequence.

To better simulate a real culture, containing background non-target DNA, an artificial culture gDNA extract was created, by mixing gDNA extracted from an isolated strain of *Marinomonas* sp. and linearized plasmid containing the *D. grandis* 18S target sequence. The copy number of the target 16S sequence was estimated from the full genome of *Marinomonas* sp. MWYL1 (U.S. D.O.E. Joint Genome Institute 2007). The rrndb database [19] gives eight 16S rRNA genes per genome for this species. We calculated that with a target sequence ∼550 bp long this would mean that in one bacterial cell there would be approximately 4400 bp of 16S sequence that would be amplified by the assay. Given that the genome size of *Marinomonas* sp. MWYL1 is 5.1 Mb, the proportion of the genome (and therefore the extracted gDNA samples) that is target sequence is estimated to be ∼0.001%.

Both the *Marinomonas* gDNA and 18S-containing linearized plasmid samples were diluted such that the amount of target sequence in both was 0.2 ng per reaction. From the target sequence:genome size estimations, this was calculated to correspond to a total culture template amount of 100 ng. This template amount was selected as it represents the amount present in 2 µl of a typical gDNA sample. As before, assays were done using template with known percentages of 16S and 18S target sequences. Assays were done in triplicate. The means, standard errors and plot of relative band brightness versus percentage target sequence were calculated using Excel 2004 (Microsoft Corporation).

### Statistical Verification of the Assay

The assay was conducted in triplicate on six separate 50 ml cultures (three *D. grandis* and three *S. diplocostata*). Genomic DNA was extracted from each culture using a CTAB-based protocol as before. Three samples from each gDNA extract were used as template in identical PCRs. Reactions were conducted separately to ensure robustness to machine error. Relative measurements of band brightness were analysed using a REML analysis to account for any random variation between gels using Genstat 10 (VSN International, UK).

### Validity of Assay for use with cDNA

The assay was conducted with cDNA made from total culture RNA and the results compared with gDNA extracted from the same culture. 100 ml of six separate cultures (three *D. grandis*, three *S. diplocostata*) were homogenized by gentle shaking. Each culture was then split into two 50 ml aliquots. gDNA was prepared from one aliquot using CTAB buffer protocol and resuspended in 30 µl of ddH_2_O. RNA was extracted from the second aliquot using a TRIzol (Invitrogen) based protocol [26]. All gDNA contamination was removed from the RNA preparation using TURBO DNase (Ambion) with the accompanying buffer and 50 mM EDTA as stop solution. The DNA-free RNA was then used for reverse transcription. The cDNA was made using random hexamer primers and Superscript III First-Strand Synthesis reverse transcriptase (Invitrogen), producing a total volume of 20 µl cDNA. 2 µl of either cDNA or gDNA was then used as template for the assay. This assay was performed in triplicate in separate reactions for gDNA and cDNA from each culture. The resulting relative band brightness measurements were analysed with a REML mixed effects model. Following adjustment of the cDNA measurements re-analysis was done using a nested ANOVA. All statistical tests were done using Genstat 10 (VSN International, UK).

### Dilution Effect Testing

Three gDNA and three cDNA samples (see above) were diluted 10-fold and 100-fold in ddH_2_O, and 2 µl used as template for the assay. Single-stranded cDNA concentrations and double-stranded gDNA concentrations were measured using a ND-1000 Spectrophotometer and ND-1000 v3.3.0 Software (NanoDrop) to verify dilution factors. The measured concentrations of undiluted samples ranged from 3500 ng/μl to 15 ng/μl. Assays were performed in triplicate in separate reactions. The resulting average 16S band brightnesses were plotted against log_10_ dilution factor. Trend lines were fitted onto this plot by linear regression and the slopes of these trend lines calculated.

### Application of Assay to Culture Purification

The assay was applied to determining the efficiency of purification by treating cultures of *D. grandis* and *S. diplocostata* with antibiotics, then filtering the treated cultures. All cultures used for purification were initially cultured in large (>50 ml) flasks, homogenized by gentle shaking before being aliquoted into sterile 50 ml Falcon tubes. Light microscope observations found that a combined treatment of 2.4 ng/ml Ampicillin (Sigma), 1.2 ng/ml Streptomycin-Penicillin (Gibco) and 1.2 ng/ml Kanamycin (Sigma) acted to reduce the numbers of bacteria present in the cultures while leaving the choanoflagellate population relatively intact. Aliquots were treated with antibiotics for 36 hours. During treatment the aliquots were cultured at 13.5°C.

Cultures were filtered through 10 µm pore size Nitex nylon mesh (Small Parts Inc, Florida USA) attached to a 3 cm bore plastic tube. This pore size allowed through bacterial cells but not loricate choanoflagellate cells of either species. Filtration was done under gravity flow. Filtration time was reduced by occasionally pipetting clear the mesh pores using a cut-off P1000 tip. The wider bore of the pipette tip reduced damage to the choanoflagellate cells or loricae. After 40 ml of culture had filtered through into a 50 ml Falcon tube, 15 ml of sterile artificial seawater (recipe as per culture conditions) was added onto the mesh. A cut-off P1000 pipette tip was used to remove the residue into a 50 ml Falcon Tube. The gDNA extracted from the residues and filtrates were assayed in comparison with gDNA from a control (untreated, unfiltered) 50 ml aliquot of the same culture.
